# Does the leading pharmaceutical reform in China really solve the issue of overly expensive healthcare services? Evidence from an empirical study

**DOI:** 10.1371/journal.pone.0190320

**Published:** 2018-01-16

**Authors:** Yunzhen He, Guanshen Dou, Qiaoyun Huang, Xinyu Zhang, Yingfeng Ye, Mengcen Qian, Xiaohua Ying

**Affiliations:** Department of Health Economics, School of Public Health, Fudan University, Shanghai, China; UNAIDS, GUYANA

## Abstract

**Background:**

Healthcare system reform of Sanming city has become a leading healthcare reform model in China. It has developed a rigorous pharmaceutical reform consisted of the Zero Mark-up Drug Policy and the Centralized Procurement of Medicine Policy to bring down drug expenses and total health expenditures. However, despite the credit and much attention have been given to Sanming’s pharmaceutical reform, its impact still remains unclear. Therefore, the purpose of this study was to explore the impact of the pharmaceutical reform of Sanming on both drug and total health expenditures.

**Methods:**

Interrupted time series analysis with three segments divided by two intervention points was employed to evaluate the impact of the pharmaceutical reform. Segment 1 was the pre-reform period which captured the baseline information. Segment 2 occurred after the first intervention point when the Zero Mark-up Drug Policy was implemented, whereas Segment 3 was after the implementation of the Centralized Procurement of Medicine Policy. Primary outcomes are outpatient drug expenditure, outpatient total health expenditure, inpatient drug expenditure, and inpatient total health expenditure. Data spanning from May 2012 to May 2014 are included.

**Results:**

Both drug and total health expenditures exhibited rising trends before any policy was carried out. The launch of Zero Mark-up Drug Policy led to significant instant reductions in levels of outpatient drug expenditure (coefficient = -6,602.99, p<0.01), outpatient total health expenditure (coefficient = -9,958.58, p<0.05), inpatient drug expenditure (coefficient = -7,520.90, p<0.01), and inpatient total health expenditure (coefficient = -16,737, p<0.01). Moreover, the previous upward trends were changed into downward trends for inpatient drug expenditure (coefficient = -2,747.02, p = 0.00) and total health expenditure (coefficient = -3,069.29, p = 0.12). However, after the implementation of Centralized Procurement of Medicine Policy, we observed no significant instant level changes and also, the inpatient drug expenditure (coefficient = 372.95, p = 0.01) and total health expenditure (coefficient = 788.76, p = 0.06) resumed upward trends again.

**Conclusions:**

Although the pharmaceutical reform could control or reduced drug expenditure and total health expenditure in short term, expenditures gradually resumed growing again and reached or even exceeded their baseline levels of pre-reform period, indicating the effect became weakened or even faded out in long term. In all, the pharmaceutical reform as a whole failed to meet its goal of combating sharp growth of drug and total health expenditure.

## Introduction

The astronomical growth of drug expenditures as well as the resulting increase of total health expenditures has imposed great burdens to patients and become a major concern in China government’s mind. Traditionally, public hospitals own by China’s governments are funded through a variety of channels, which can be broadly categorized into three groups namely, government subsidy, drug revenue, and medical service fees [1]. Since China’s 1978 Reform and Opening-up policy, the proportion of government’s direct subsidies to public hospitals have been significantly declined annually. As of 2009, the proportion of government subsidy out of total hospital revenue was merely 10% [2]. As a way to balance the financial loss due to reduced government subsidy for hospitals, the drug mark-up policy was put forward which allows hospitals to set a markup up to 15% on the wholesale prices of drugs [3]. Facing drug markup of 15% and driven by financial incentives, physicians tend to prescribe expensive drugs or over-prescribe unnecessary drugs to patients in order to gain profits [4] and hence, made drug revenue a major funding source for public hospitals. Drug expenditure per capita grew rapidly from 36.59 Yuan to 467.04 Yuan between 1990 and 2008, at an annual growth rate of 15% which outran the growth rate of GDP [5]. In 2008, drug expenditure accounted for as high as 42.67% of total health care expenditure [6]. The profit-driven behavior and its resulting irrational use of medicines were recognized as one of the influential factors of the drastic increase in drug expenditures and have contributed to serious economic burdens for patients, especially for inpatients in rural areas [7]. In order to combat sharp growth of total health expenditure, China’s central government decided to cut off the profit chain between health providers and pharmaceuticals by eliminating the drug markups. In the year of 2009, China central government officially issued a new round of healthcare system reform, which required that the additional markup should be gradually removed from drug sales in public hospitals [8].

Following that, Sanming city embarked on its comprehensive healthcare system reform in 2012 and has successfully become one of the most leading healthcare reform models in China [9, 10, 11]. The “Sanming model” consisted of reforms in health insurance, pharmaceutical and health institution sectors in general. For the pharmaceutical reform, it was mainly made up of the Zero Mark-up Drug Policy (hereinafter ZMDP) and the Centralized Procurement of Medicine Policy (hereinafter CPMP) [12]. ZMDP, introduced in February 2013, was designed to remove mark-ups for any drug sold by public hospitals in order to alleviate hospitals’ heavy reliance on drug sales. In this way, public hospitals were only allowed to prescribe drugs at their wholesale prices. Meanwhile, the loss in drug revenues was allowed to be offset through higher service fees and government subsidies. After the implementation of ZMDP, Sanming government quickly rolled out CPMP in June 2013 to further bring down drug prices. The core philosophy behind CPMP was to strengthen bargaining power on the demand’s side and streamline supply chains of medicines. Distinguished from the traditional way where individual public hospital was seen as one single buying unit, Sanming government unified health administrative department, all health insurance schemes and public hospitals into just one drug procurement leading group to increase its negotiating power to bring down drug prices. Moreover, “Two Invoices” system was introduced to reduce unnecessary intermediate medicine distributors, where only drugs with two produced invoices can be covered by health insurance, one from manufacturer to the distributor and the other one from the distributor to the hospital [12]. The supply chain can be shortened by “Two Invoice” system and hence, a more transparent medicine procurement process and large savings would be generated.

However, it still remains unclear whether the implementation of the pharmaceutical reform has yield intended results such as bringing down drug expenditures in Sanming city. In spite of well recognition of the “Sanming model” by China central government and some academicians, to our best knowledge, no empirical studies with either rigorous experimental or quasi-experimental design examining the effect of the pharmaceutical reform in Sanming have been found. Currently preliminary research studying the effect of the pharmaceutical reform in Sanming primarily focused on either drug prices or drug expenses [12], not total health expenditures. However, we found it necessary to also incorporate total health expenditures in evaluation of the effect of the pharmaceutical reform since total health expenditures is where the ultimate goal of any pharmaceutical policy lies in. The purpose of this present article is to, for the first time, comprehensively explore the impact of the pharmaceutical reform on both drug expenses and total health expenditures in Sanming city with rigorous quasi-experimental design.

## Methods

### Study design

Our study aims to explore the impact of the overall pharmaceutical reform by taking a detailed look at its two components, ZMDP and CPMP. In February 2013, Sanming city implemented ZMDP, which is regarded as the first intervention. Following ZMDP, CPMP was rolled out 4 months later and represents the second intervention in this study. Interrupted time series analysis with two intervention points is employed to estimate both the transient (the level change in outcomes) and long-term (the change in trends of outcomes) effects of these two policies on drug expenditure and total health expenditure [13].

### Outcome variables and data sources

Outcome measures are defined as outpatient drug expenditure, inpatient drug expenditure, outpatient total health expenditure and inpatient total health expenditure for each month, respectively. It is necessary to mention here that drug expenditure account for a portion of total health expenditure. Besides drug expenditure, total health expenditures also consist of examination fees, test fees, surgery fees and so on. Data of outcome variables were derived from hospital financial statements that were publicly disclosed on the “Health Sanming” online platform on a monthly basis. A total of 24 monthly time points from May 2012 until May 2014 were collected, which is sufficient enough to conduct an interrupted time series analysis [13].

### Statistical analysis

Interrupted time series design is the strongest, quasi-experimental approach for evaluating longitudinal effects of interventions [13]. A segmented regression model, with two intervention points in February 2013 and June 2013 respectively, is specified as followed:

$$Y_{t}=\beta_{0}+\beta_{1}*\;{time}_{t}+\beta_{2}*\;{ZMDP}_{t}+\beta_{3}*\;{time\;after\;ZMDP}_{t}+\beta_{4}*\;{CPMP}_{t}+\beta_{5}*\;{time\;after\;CPMP}_{t}+e_{t}$$

Where *Y*_*t*_ is the outcome variable in time t; *time* is a continuous variable counting the number of months at time t from the start of the observation period; ZMDP is an indicator for time t occurring before (ZMDP = 0) or after (ZMDP = 1) the zero mark-up drug policy, which came into effect at month 10 in the series; *time after ZMDP* is a continuous variable counting the number of months at time t from the start of the implementation of ZMDP, which was set at 0 before ZMDP; CPMP is an indicator for time t occurring before (CPMP = 0) or after (CPMP = 1) the centralized procurement of medicine policy that was implemented at month 14 in the series; *time after CPMP* is a continuous variable counting the number of months at time t from the start of the implementation of CPMP, which was coded 0 before CPMP and (time—13) after CPMP; *e*_*t*_ is an error term at time t. For parameters, *β*_0_ estimates the baseline level of the outcome at time zero; *β*_1_ captures the time trend before any policy took place, namely the baseline trend; *β*_2_ (*β*_4_) estimates the change in the value of the outcome immediately after the implementation of ZMDP (CPMP), compared with the value of the outcome at the end of the preceding segment; *β*_3_ (*β*_5_) estimates the change in the trend in the outcome after the implementation of ZMDP (CPMP), compared with the monthly trend in the preceding segment, where the trend represents the rate of change of the outcome variable, namely the slope. Thus *β*_1_+*β*_3_ is the post-ZMDP slope while *β*_1_+*β*_3_+*β*_5_ represents the post-CPMP slope. In this study, Wald test was employed to examine the statistical significance of combined effects of coefficients.

Several diagnostic tests were employed to assess the validity of segmented regression model. First of all, Dickey–Fuller test showed that there existed seasonal fluctuations and hence, quarterly seasonal dummy variables were incorporated to control for the issue of seasonality to avoid spurious associations [14]. Durbin–Watson test for regression with a full set of seasonal dummy variables suggested the existence of first–order autocorrelation [15]. Therefore, autoregressive integrated moving average method (“ARIMA”) with first–order autoregressive procedure (“AR(1)”) was used to correct for autocorrelation and estimate regression parameters [16, 17]. Moreover, Breusch–Pagan statistic was estimated and the result indicated of existence of heteroscedasticity for both outpatient drug and total health expenditures and thus, we used robust regression to obtain robust variance estimators for expenditures in outpatient sector. Lastly, the estimate of Kolmogorov-Smirnov statistic suggested that residuals conformed to normal distribution.

## Results

Sanming is a city of roughly 2.7 million populations located in Fujian Province and covers an area of 22,900 square meters. With a GDP per capita of US$7,200, Sanming is economically middling by Chinese standard [9]. As shown in Table 1, it accommodates a total of 25 county level and above public hospitals, among which there are 22 general hospitals (traditional Chinese medical hospitals included) and 3 specialty hospitals. Of 22 general hospitals, there are 4 tertiary hospitals with designated bed capacity of over 500 beds, the rest of the general hospitals are secondary hospitals containing beds ranging between 200 and 500.

**Table 1:** Brief profile of public hospitals in Sanming city.

Hospital Characteristics	Number	Proportion
By Hospital Level		
Tertiary	4	16%
Secondary	21	84%
By Types of Hospitals		
General (TCM* included)	22	88%
Specialized	3	12%
By Designated Bed Capacity		
≥1000	1	4%
500≤x<1000	4	16%
200≤x<500	10	40%
<200	10	40%

* TCM are short for traditional Chinese medical hospitals

### Monthly drug expenditures

**Fig 1:**
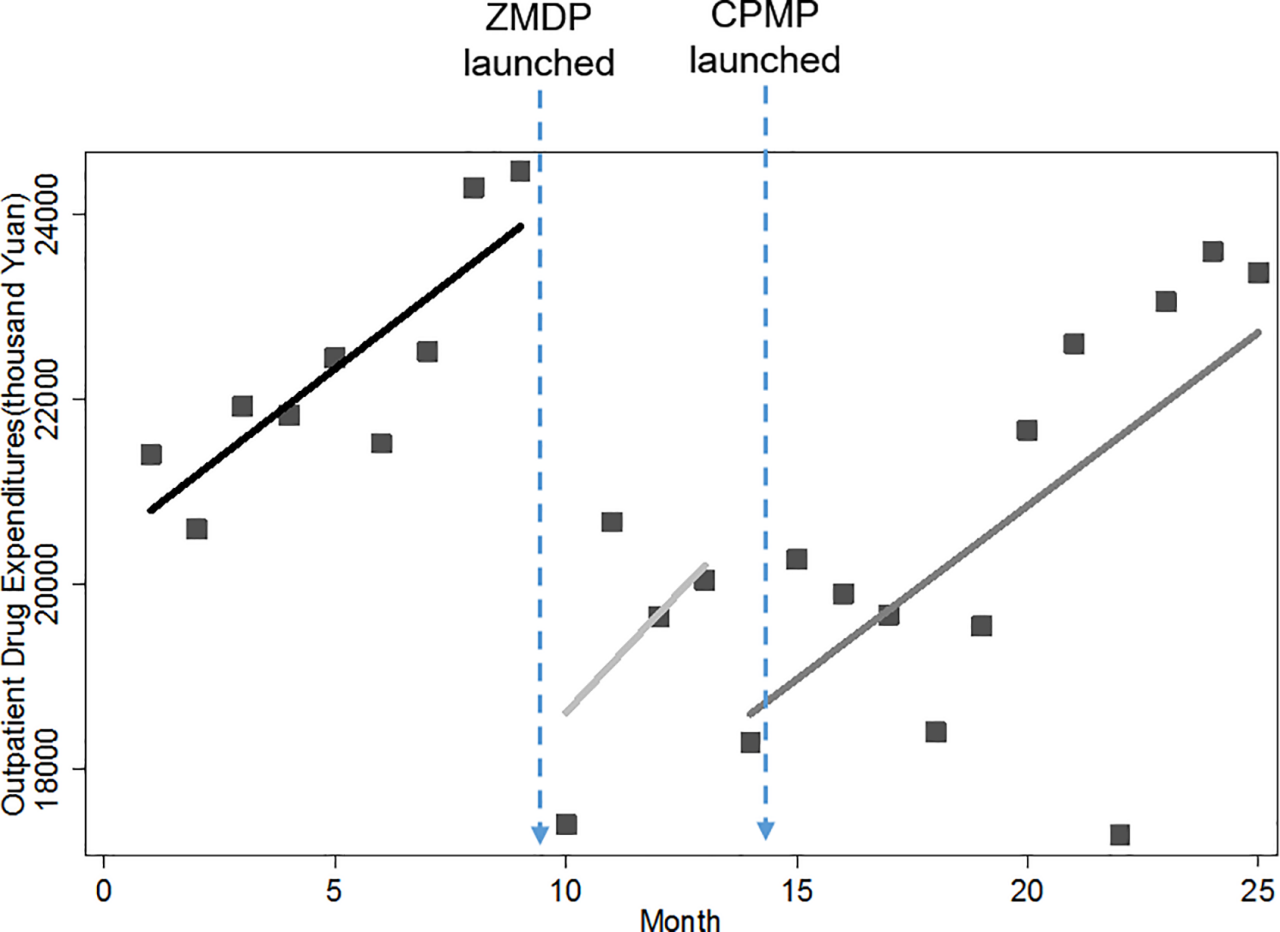
Trend in the monthly outpatient drug expenditure for public hospitals in Sanming city.

**Fig 2:**
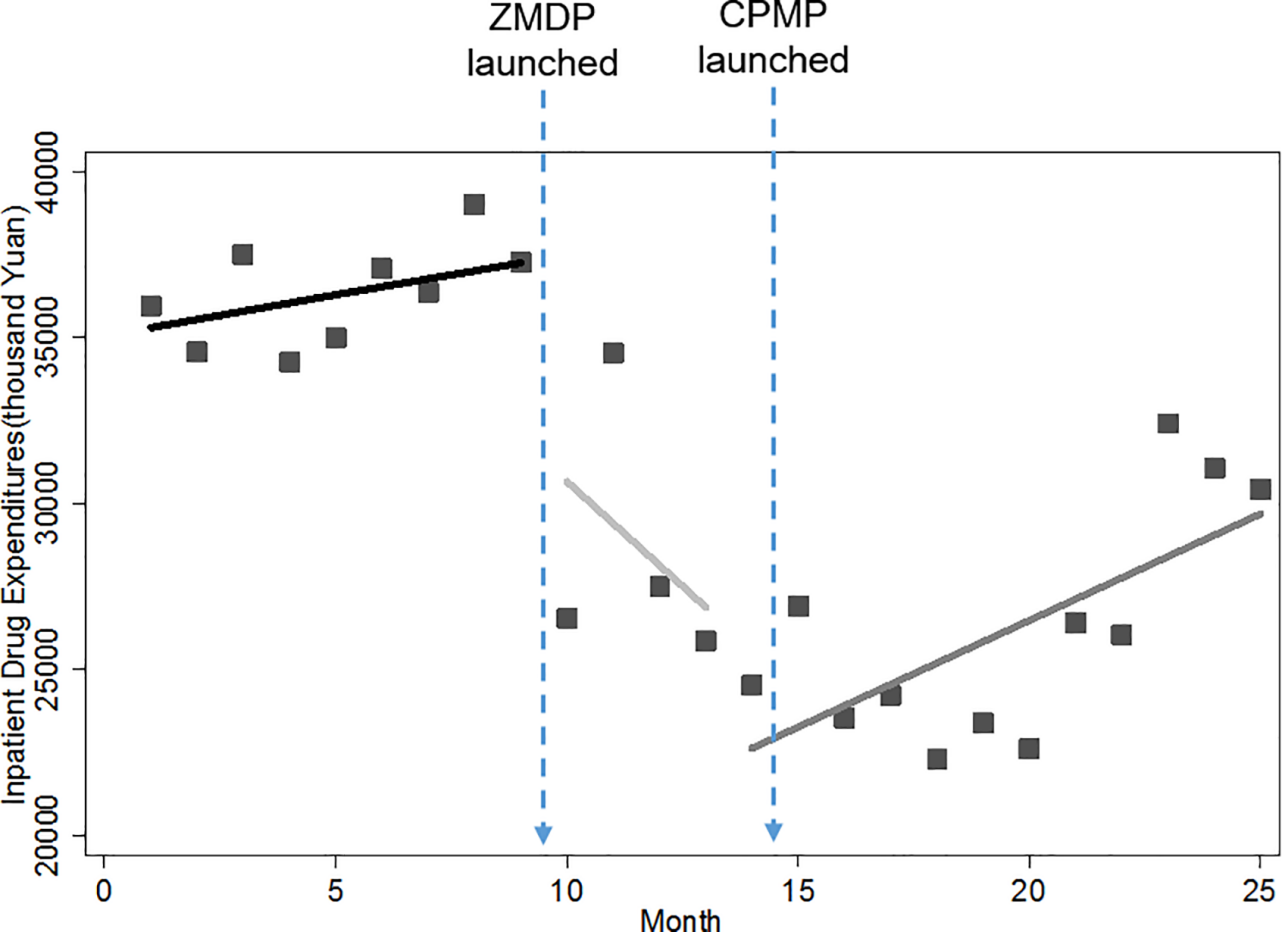
Trend in the monthly inpatient drug expenditure for public hospitals in Sanming city.

As shown in Figs 1 and 2, there are two significant interruption points and the resulting three segments where different changing patterns of drug expenditure were displayed. First of all, increasing trends in monthly drug expenses were observed for both outpatient and inpatient services before the introduction of the pharmaceutical reform. Immediately after the launch of ZMDP, we found instant drops in outpatient and inpatient monthly drug expenditure. We noted that the trend of outpatient drug expenditures remained increasing regardless of the implementation of the ZMDP or CPMP. However for inpatient services, the previous increasing trend of inpatient drug expenditure was turned into a declining trend. The declining trend persisted for a short period of 5 months until the introduction of CPMP, after which the increasing trend resumed. Despite growing again, it is worth mentioning that the level of inpatient drug expenditure has been dropped dramatically after the ZMDP and CPMP, to the point where it was far less than its baseline level.

Table 2 further provides numerical details of level and trend changes related to ZMDP and CPMP. Before the ZMDP, there were month-to-month increases of 589.69 (p<0.01) and 962.30 (p<0.01) thousand Yuan in outpatient and inpatient drug expenditure with statistically significant, respectively. Note that significantly instant level change occurred right after the ZMDP, in which outpatient and inpatient drug expenditure decreased by 6,602.99 (p<0.01) and 7,520.90 (p<0.01) thousand Yuan, respectively. There was significant change in the trend of inpatient drug expense. The month-to-month increase of 962.30 thousand Yuan was inversed to a statistically significant decrease of 2,747.02 thousand Yuan (p<0.05) month-to-month. However, after the CPMP was launched, this downward trend in drug expenses gave place to an upward trend of 372.95 thousand Yuan (p<0.01) with statistical significance. Note that the later increasing trend of 372.95 thousand Yuan was slower than that of 962.30 thousand Yuan in baseline, indicating of slowing growth rate after the CPMP. Different from inpatient sector, there was only decline in growth rate rather than inversion occurring to the trend of the outpatient drug expenditure after the pharmaceutical reform, but without statistical significance.

**Table 2:** The impact of the implementation of ZMDP and CPMP on monthly outpatient and inpatient drug expenditures (thousand Yuan).

Parameter	Coefficient (for outpatient drug expenditures)		Coefficient (for inpatient drug expenditures)	
Intercept *β*_0_	21,209.34^***^ (446.43)		35,782.55^***^ (837.05)	
Baseline trend *β*_1_	589.69^***^ (112.15)		962.30^***^ (137.15)	
Level change after ZPMP *β*_2_	- 6,602.99^***^ (751.45)		- 7,520.90^***^ (2413.46)	
Trend change after ZMDP *β*_3_	- 602.80 (501.94)		- 3,709.32^***^ (754.41)	
Level change after CPMP *β*_4_	994.63 (1,083.54)		1,492.11 (1,731.59)	
Trend change after CPMP *β*_5_	217.92 (375.72)		3,119.97^***^ (720.54)	
Summer	- 1,565.04^***^ (506.32)		-3,030.21^**^ (1198.55)	
Autumn	- 2,628.48^***^ (519.51)		-5,902.38^***^ (889.60)	
Winter	- 1,920.55^*^ (1,006.08)		-5,749.70^***^ (985.27)	
**Combined Effect**				
	Coefficient	P-value	Coefficient	P-value
*β*_1_ + *β*_3_	- 13.11	0.97	- 2,747.02	0.00
*β*_1_ + *β*_3_ + *β*_5_	204.81	0.01	372.95	0.01

Note: 1) P-values:

*** p<0.01

** p<0.05

* p<0.1

2) the combined effect was examined by the Wald test

3) standard error in parentheses

### Monthly total health expenditures

**Fig 3:**
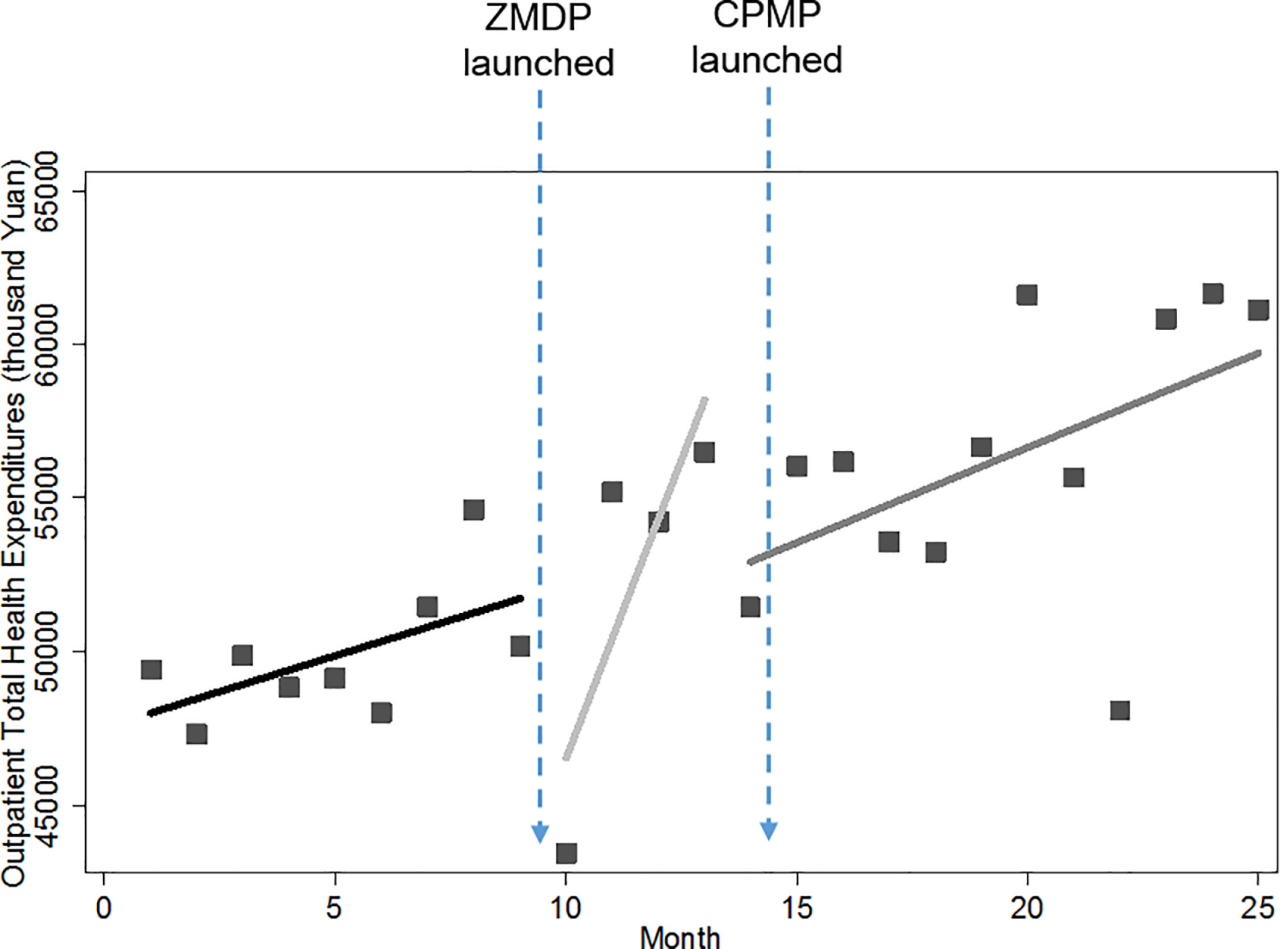
Trend in the monthly outpatient total health expenditures for public hospitals in Sanming city.

**Fig 4:**
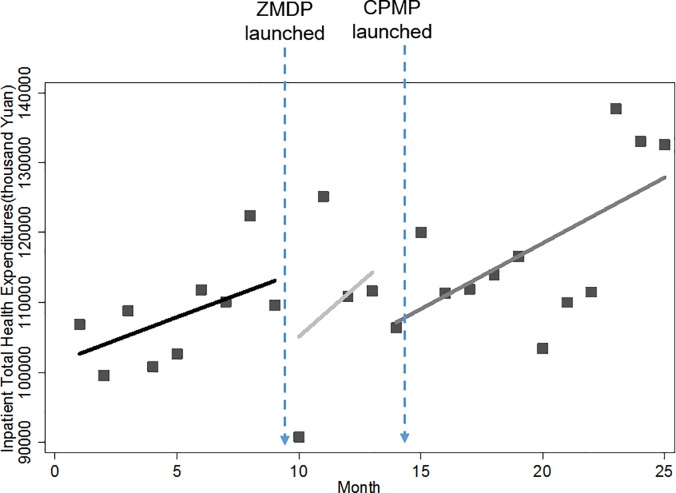
Trend in the monthly inpatient total health expenditures for public hospitals in Sanming city.

Figs 3 and 4 showed pictures of how the level and trend of total health expenditures are changed by the pharmaceutical reform. Similarly, a rise in baseline trend and an instant drop after the implementation of ZMDP were also found in both outpatient and inpatient total health expenditures. However, it seems like the pharmaceutical reform didn’t have too much impact on total health expenditures since expenditures remained growing throughout the entire study period and compared with baseline levels, the levels of both outpatient and inpatient total health expenditures were even higher at the end of the study period.

Table 3 below further examined numerically the impact of the ZMDP and CPMP on total health expenditures. Before the ZMDP, outpatient and inpatient total health expenditures increased at the rate of 1,001.09 (p<0.05) and 3,821.82 (p<0.01) thousand Yuan month-to-month, respectively. Significant instant level changes were noted immediately after implementation of the ZMDP. The outpatient total health expenditure dropped sharply by 9,958.58 thousand Yuan (p<0.01) while its trend remained increasing. Unlike outpatient sector, the inpatient total health expenditure was not only dropped by 16,737.14 thousand Yuan (p<0.05) instantly, but also its previous growing rate dropped dramatically by 6,891.11 thousand Yuan (p<0.01), to the extent that the rising trend was inversed to a falling trend though without statistical significance (p = 0.12). Note that this decreasing trend wasn’t displayed in Fig 4 due to seasonal fluctuations. Contrary to expectations, the launch of the CPMP did not present any controlling effect on inpatient total health expenditures. The level of inpatient total health expenditures went up significantly by 16,018.12 thousand Yuan (p<0.01) immediately after the CPMP. Besides, its falling trend was replaced by the monthly increase of 788.76 thousand Yuan (p = 0.06). For outpatient sector, the rising trend of total health expenditures still remained but was just reduced by 1,864.55 thousand Yuan (p<0.05) to a monthly increase of 180.83 thousand Yuan after the CPMP.

**Table 3:** The impact of the implementation of ZMDP and CPMP on monthly outpatient and inpatient total health expenditures (thousand Yuan).

Parameter	Coefficient (for outpatient total health expenditures)		Coefficient (for inpatient total health expenditures)	
Intercept *β*_0_	49,744.84^***^ (1,702.21)		105,100.50^***^ (1982.98)	
Baseline trend *β*_1_	1,001.09^**^ (418.92)		3,821.82^***^ (401.95)	
Level change after ZPMP *β*_2_	- 9,958.58^***^ (2,652.92)		- 16,737.14^**^ (7,353.92)	
Trend change after ZPMP *β*_3_	1,044.29 (1,316.16)		- 6,891.11^***^ (2,065.77)	
Level change after CPMP *β*_4_	- 2,347.80 (3,433.73)		16,018.12^***^ (4,643.88)	
Trend change after CPMP *β*_5_	- 1,864.55^**^ (903.57)		3,858.05^*^ (2,069.80)	
Summer	- 4,608.35^**^ (1,925.07)		- 14,122.70^***^ (3,433.26)	
Autumn	- 6,020.82^***^ (2,183.33)		- 18,053.04^***^ (2,707.81)	
Winter	- 6,018.37^**^ (2,880.29)		- 23,965.53^***^ (1,982.98)	
**Combined Effect**				
	Coefficient	P-value	Coefficient	P-value
*β*_1_ + *β*_3_	2,045.38	0.04	- 3069.29	0.12
*β*_1_ + *β*_3_ + *β*_5_	180.83	0.56	788.76	0.06

Note: 1) P-values

*** p<0.01

** p<0.05

* p<0.1

2) the combined effect was examined by the Wald test

3) standard error in parentheses

## Discussion

“Sanming model” is currently one of the leading healthcare reform models in China and well recognized by the central government and World Bank [18, 19]. The pilot trials in Sanming are supposed to be scaled-up in the next few years. Despite the credit given to Sanming comprehensive healthcare reform, our study take a specific look at its pharmaceutical reform and present a different story where in general its pharmaceutical reform only exerted limited effect in terms of curbing exponentially growing drug expenses and total health expenditures.

Study results showed that the pharmaceutical reform as a whole was able to successfully either bring down drug and total health expenditures or curb their growth rates in short term. However, the effect became weakened or even faded out in long term. Both drug and total health expenditure levels were dropped sharply after the launch of the ZMDP, especially for inpatient sector, where the previous rising trends of drug and total health expenditures were even changed into decreasing trends. Unfortunately, drug and total health expenditures increased month-to- month again after the CPMP and even worse, exceeded their baseline levels except for inpatient drug expenditures, suggesting the impact of the ZMDP was gradually faded out in long term. Currently published studies presented a mixed result regarding the long term effect of the ZMDP [5, 20, 21]. One possible explanation is that previous studies of the impact of the ZMDP mainly focused on primary health institutions and the scope was restricted in national essential drugs. But the ZMDP in Sanming was expanded to any drug sold there and all levels of health institutions including tertiary, secondary and primary hospitals. Therefore, different scopes of policy may yield different study results. Another potential reason may be due to the drug kickbacks. Kickbacks are bonus that healthcare companies usually pay to physicians under the table to boost product sales. In China, kickbacks wildly exist and are accepted by physicians as a way to compensate for their medical professions. Therefore although mark up on drug prices has been removed and hospitals are not able to earn revenues through drug consumptions any more, physicians still have incentives to over-prescribe drugs to patients. [22]. While the increase in drug expenses after the Sanming’s reform is counter-intuitive, the growth of total health expenditure is possible since hospitals may increase costs of other services (e.g. diagnostics, surgeries, inpatient room rates etc.). And also, physicians may seek to over-provide excessive services such as unnecessary examinations and tests to generate revenues in order to compensate the loss in drug revenues [20, 21, 23]. As a result, the total health expenditure remained growing. This study finding regarding the increase in total health expenditures suggests that further study into change in physician behavior or use of other services is warranted.

Another discovery was that compared with the CPMP, the implementation of ZMDP was more effective in reducing drug expenses and total health expenditures. According to statistical analysis, the launch of ZMDP not only led to reduction in levels of drug and total health expenditures, but also controlled their growth rates. This is consistent with currently available studies [5, 21, 24, 25], which also showed that the ZMDP would lead to great reduction drug expenditures. The reason behind is straightforward since the ZMDP directly reduced drug retail prices by cancelling drug mark-ups on wholesale prices and therefore, the decline in prices would further lead to drug revenues. However, there weren’t any significant reductions occurring in drug and total health expenditures after CPMP. Contrarily, inpatient drug and total health expenditures returned to growth from their decreasing trends of the preceding period. This result revealed that the CPMP failed to either reduce the drug expense or combat its growth. This discovery was also in line with currently available studies [22,23,26], where the CPMP was reported to be insufficient not only to lower drug wholesale prices but also to bring down drug expenses. The failure of CPMP may be justified by the fact that health institutions had limited incentives to lower their purchase prices (wholesale prices) to save money because of the ZMDP [26]. Moreover, centralized drug procurement system has been in place for more than a decade and the CPMP launched in Sanming city was nothing more than a new version of it and therefore, it is reasonable to believe that pharmaceutical companies has already adapted to the CPMP well.

Furthermore, out study results displayed the fact that the ZMDP had differential impact on expenditures between outpatient and inpatient sector. Changes in levels and trends of expenditures were observed for inpatient sector, whereas for outpatient sector, only changes in levels were reported and their increasing trends seemed to be immune from regulation and still persisted after the ZMDP. And also, although trends returned to growing after the CPMP, the level of inpatient drug expenditure was far below its baseline level, which was contrary to the outpatient sector where drug expenditure has almost reached its baseline level. Such differential effect was also reported in another study where reductions in drug expenditures per discharged patient for inpatient care were statistically significant while outpatient drug expenditures per visit not [27]. However, the reason behind this remained unclear, indicating the necessity of further research.

## Conclusion

Interrupted time series analysis was conducted to estimate the short term and long term effect of the pharmaceutical reform in Sanming city. Generally speaking, the pharmaceutical reform as a whole failed to meet its goal of combating sharp growth of drug and total health expenditures. In particular, the pharmaceutical reform could control or reduced expenditures in short term, but the effect became weakened or even faded out in long term, indicating that drug and total health expenditures gradually returned to rise again. This result revealed that interventions such as ZMDP/CPMP designed to reduce cost by cutting hospital margins may not work since margins could be made up from elsewhere. Therefore, interventions targeting structural reduction in costs may work better. Our study also found that the ZMDP were more effective than the CPMP in combating expensive health care services but its impact only persisted for a short period of time. In all, more scientifically designed policies are needed to prevent the drug and total health expenditures from continuing to rise again.
